# Improved RANSAC Point Cloud Spherical Target Detection and Parameter Estimation Method Based on Principal Curvature Constraint

**DOI:** 10.3390/s22155850

**Published:** 2022-08-05

**Authors:** Qinghua Wu, Jiacheng Liu, Can Gao, Biao Wang, Gaojian Shen, Zhiang Li

**Affiliations:** Hubei Key Laboratory of Modern Manufacturing Quantity Engineering, School of Mechanical Engineering, Hubei University of Technology, Wuhan 430068, China

**Keywords:** 3D point cloud, spherical target detection, sphere parameter estimation, RANSAC, large-scale combined measurement

## Abstract

Spherical targets are widely used in coordinate unification of large-scale combined measurements. Through its central coordinates, scanned point cloud data from different locations can be converted into a unified coordinate reference system. However, point cloud sphere detection has the disadvantages of errors and slow detection time. For this reason, a novel method of spherical object detection and parameter estimation based on an improved random sample consensus (RANSAC) algorithm is proposed. The method is based on the RANSAC algorithm. Firstly, the principal curvature of point cloud data is calculated. Combined with the k-d nearest neighbor search algorithm, the principal curvature constraint of random sampling points is implemented to improve the quality of sample points selected by RANSAC and increase the detection speed. Secondly, the RANSAC method is combined with the total least squares method. The total least squares method is used to estimate the inner point set of spherical objects obtained by the RANSAC algorithm. The experimental results demonstrate that the method outperforms the conventional RANSAC algorithm in terms of accuracy and detection speed in estimating sphere parameters.

## 1. Introduction

During the maintenance of aircraft landing gear, it is necessary to detect assembly errors, such as parallelism of the front and rear axles of the frame and axiality between the shock absorber prop and sleeve. Because of the sizeable measurement space range and the complex structure of measured parts, they need to be measured by a combined measurement method. Combined measurement is a method to select a measurement method with large-scale precision characteristics to achieve global measurement and control and to select a precise and an efficient morphology measurement method as the terminal measurement to collect high-density point clouds [1,2]. Its key principle is to transform the terminal scan data from different locations into a unified global coordinate reference system with the help of auxiliary targets [3]. Three-dimensional structured light scanning technology is often used as the terminal measurement device in combined measurements due to its good collimation, non-contact mode, high accuracy, and fast measurement [4,5].

Because the sphere is used as the target of detection, any error of the sphere center that it determines will be propagated as an error in the coordinate system transformation. In order to improve the efficiency and accuracy of coordinate system unification during measurement, many studies have been devoted to improving the sphere detection accuracy and shortening the calculation time. Three-dimensional point cloud sphere detection includes the 3D Hough transform [6], registration [7], random sample consensus (RANSAC) [8], region growing [9], and other methods. For example, Schnabel et al. [10] proposed the RANSAC method for 3D sphere detection. This method can effectively detect spherical regions in a point cloud. It has high detection accuracy and can suppress the effect of noise. However, the traditional RANSAC has high computational complexity and relatively high time consumption [11]. Other researchers [12,13] have put forward some improved RANSAC methods. These improved RANSAC algorithms can effectively segment targets, but they do not delve into precise target fitting. Camurri et al. [14] applied the 3D Hough transform of three-dimensional space for sphere identification and the detection of point cloud data. It can calculate sphere parameter values from point clouds containing noise and suppress noise points. However, in practical application, the 3D Hough transform is more complicated in three-dimensional space, with large computation and high time consumption. Abizaina et al. [15] proposed a method based on the 3D Hough transform to detect spherical point clouds in 3D point cloud data generated by the Kinect sensor, and it has higher computational efficiency than traditional algorithms. Other researchers [16,17] also proposed methods to improve the sphere detection efficiency and robustness of 3D Hough detection. These methods based on the 3D Hough transform can effectively detect the sphere in the point cloud and suppress noise. However, their detection accuracy is based on the resolution of discrete space, so the calculation cost will increase sharply with the improvement of accuracy. In many improved methods, the sphere’s radius in the point cloud needs to be determined in advance, and the spherical point cloud must reach the hemisphere to be feasible. Anh-vu et al. [18] applied the region growing method for sphere detection, selected seed points to expand according to the criteria of surface membership, and divided point clouds into different surfaces through iteration. Nurunnabi et al. [19] improved the region growing method. This method sets the selection region of seed points to the area near the artificial filter edge and selects the points with the least curvature in the region as seed points. The method can detect the spherical model in the point cloud quickly, but the accuracy of detection depends mainly on the pre-selected seed points. When the scene contains many noise point clouds, it is challenging to select seed points, and the detection accuracy is low. Wang et al. [20] proposed an automatic method of laser point cloud registration based on spherical target detection, which can quickly detect spherical targets in the point cloud. Huang et al. [21] proposed a registration method based on an auxiliary spherical target combined with the ICP algorithm, and the spherical target is used to provide spherical constraints for registration. These registration methods can quickly detect spherical objects after scene segmentation, but they lack a noise suppression mechanism after scene segmentation with noise.

When 3D structured light scanning is used as a large-scale combined measurement terminal, the scanned point cloud data contain the spherical point cloud below but close to the hemisphere, environmental point, outlier point and regular point clouds of the measured object. That is, non-spherical point clouds account for a relatively high proportion of point clouds. The RANSAC algorithm can suppress the influence of point cloud noise on spherical feature recognition, and it has high robustness and efficiency. Therefore, it is suitable for large-scale measurement coordinate unification scenarios.

However, due to the random selection of sample points in the detection process of traditional RANSAC, the model established by the random selection of sample points in the presence of a lot of noise point clouds is often an error model, and the error model can only be determined after calculating the inner point set every time, which wastes a lot of time. To address this problem, Shuyang Shang et al. [22] proposed an improved RANSAC point cloud sphere detection method based on irrelevant point markers. Triangulation and statistics are used to mark irrelevant points of spherical shapes according to point cloud spacing, which cannot be regarded as sample points, thus improving the iteration efficiency. However, this method is suitable for scenes where noise only disperses the clutter. In addition to discrete outliers, there are also measured points with regular point cloud n large-scale combined measurement point cloud data, so this method is unstable for detection. There is another problem with the traditional RANSAC. The algorithm uses the least squares method to correct the parameters of the spherical model, which considers the coefficient matrix to be constant. There are errors in point cloud data, and the coefficient matrix is also affected by errors [23]. Therefore, a better-fitting result cannot be obtained when the threshold value is large.

This paper proposes an improved RANSAC method based on principal curvature constraint (PC-RANSAC) point cloud spherical target detection and parameter estimation to solve the above problems. It mainly improves the traditional RANSAC algorithm by first adding a constraint condition based on the principal curvature of the point cloud. Four points of random nearest neighbors are searched according to the k-d nearest neighbor algorithm and then constrained according to their principal curvatures to improve the sample quality. Secondly, the traditional RANSAC algorithm only considers the error in the Z direction but ignores the errors in the X and Y directions. This improved method uses the total least squares method to optimize the estimation of spherical target parameters.

## 2. Systems and Methods

### 2.1. System Design

**Figure 1:**
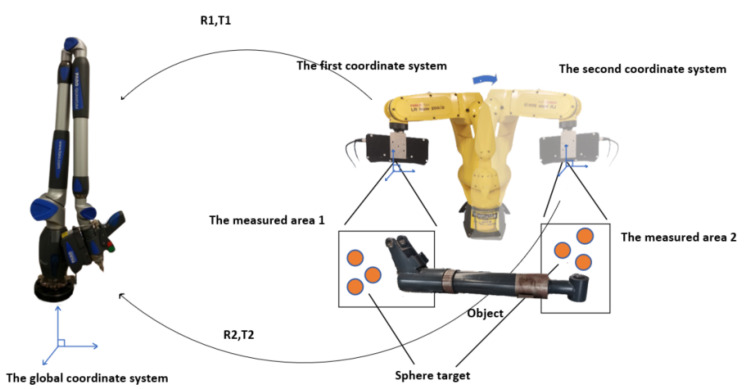
Diagram of combined measurement system.

As shown in Figure 1, the hardware of the combined measurement system includes a flexible joint arm, a 3D scanner, a manipulator, and two sets of spherical targets. The spherical target consists of multiple (at least three) ceramic standard spheres fixed in relative positions. The flexible joint arm is the global measurement coordinate system in the combined measurement system. The manipulator drives the 3D scanner as the measurement terminal to obtain the point cloud on the measured object’s surface. The two groups of spherical targets are placed in measurement areas 1 and 2 of the measured object, respectively. The position of the spherical target in the global measurement coordinate system can be measured by the flexible joint arm. During the measurement, the 3D scanner at the end of the manipulator is controlled to scan the two measurement areas. The measured element point clouds and target point clouds in the two regions are each obtained. In this way, the coordinates of the target in the global measurement coordinate system and the coordinates of the 3D scanner measurement terminal are known. Then, the point cloud data of the measured elements in the region where the spherical target is located can be converted to the global measurement coordinate system. Finally, coordinate unification of the measured elements is achieved. The key factors affecting the measurement accuracy of the system are the detection of spherical targets and the estimation of parameters. This paper elaborates on this.

### 2.2. PC-RANSAC Point Cloud Sphere Detection Algorithm

**Figure 2:**
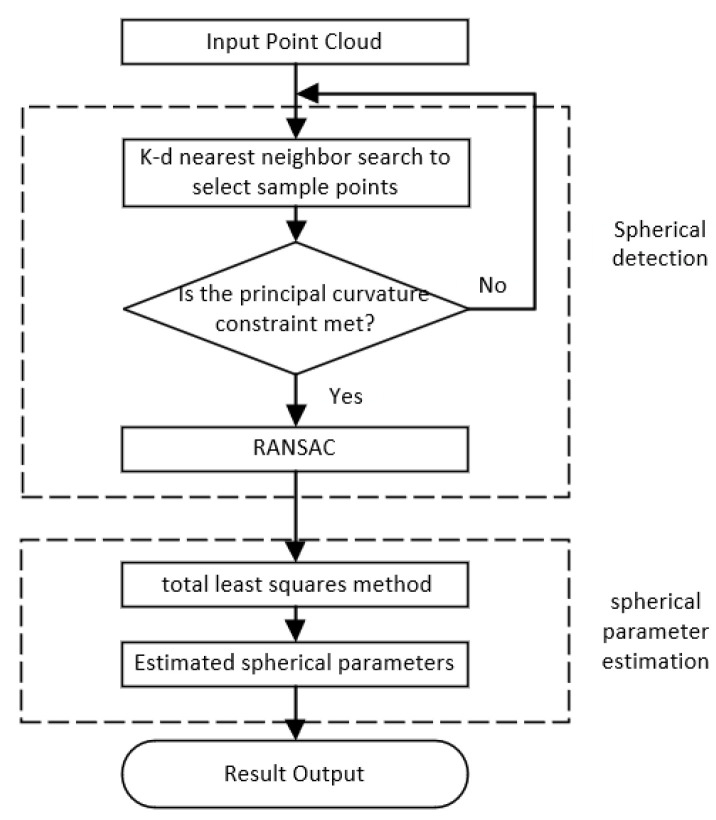
Algorithm flow chart.

The process based on the fast, robust PC-RANSAC point cloud sphere detection algorithm is shown in Figure 2. It is divided into two parts: sphere detection and sphere parameter estimation. In spherical target detection, four random neighboring points are searched as the current sample points by the K-D nearest neighbor search algorithm. Point selection is constrained by the principal curvature of the sample point. If the condition is not satisfied, a sample point is selected again to improve the quality of the sample point. In sphere parameter estimation, it considers point cloud data coefficient matrix errors and the observation vector. The total least squares algorithm optimizes the estimation of the spherical target center coordinates.

#### 2.2.1. Principal curvature constraint for sample point selection

At any point *P* on a continuous smooth surface *S*, there exist an infinite number of regular curves passing through that point that have different normal curvatures at point *P* [24,25]. The two maximum and minimum extreme values $K_{1}$ and $K_{2}$ are called the principal curvature of the point. If all points of continuous surface *S* satisfy $K_{1}=K_{2}\neq 0$, surface *S* must belong to the same sphere, and the normal curvature of all points is equal to the sphere’s radius [26,27]. If adjacent points with the same principal curvature are found, the surface constructed by these field points is the sphere in the point cloud. Let its normal unit vector at point *P* be *N* for each point *P* in the point cloud. The method to estimate the normal curvature at point *P* using point coordinates and normal phase vectors is as follows.

Let us assume that there are *m* neighboring points near *P*, $q_{i}\;$ is the ith nearest neighbor point of point *P*, and the normal vector of $q_{i}\;$ is $M_{i}$. Let the orthogonal coordinate system (X,Y,Z) be the local coordinate system *L* with point *P* as the origin, and let *N* denote the normal vector of point *P*. In *L*, *X* and *Y* are orthogonal unit vectors, and the coordinates of *P* are  (x,y,z), those of $q_{i}$ are $\left(x_{i},y_{i},z_{i}\right)$, and those of $M_{i}\;$ are $\left(n_{xi},n_{yi},n_{zi}\right)$. Let point *P* have a normal vector $N=(n_{xp},n_{yp},n_{zp})$. Suppose that the three axes *X*, *Y*, *Z* are X=(−sinϕ,cosϕ,0), Y=(cosφcosϕ,cosφsinϕ,−sinφ), and $Z=N=(n_{xp},n_{yp},n_{zp})$, where $\phi =\arctan(n_{xp}/n_{xp})$ $\phi =arc\cos(n_{zp})$.

**Figure 3:**
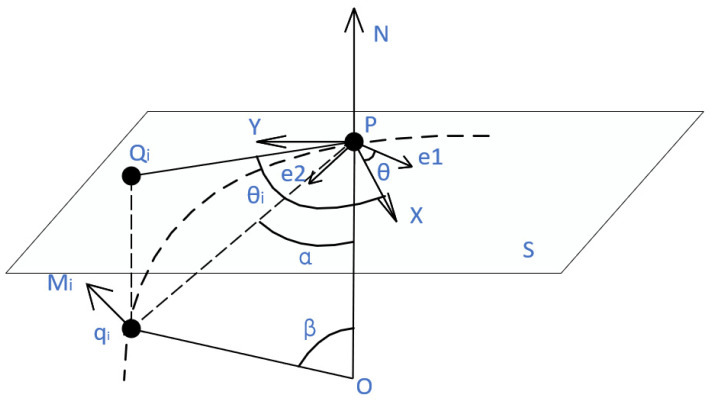
Local coordinate system L.

Then, the normal curvature $k_{n}^{i}$ of point *P* can be estimated by constructing approximate triangles using each point *P* and its normal vector, the nearest neighbor, and the normal vector of the nearest neighbor. Figure 3 shows the geometric relationships of these variables.

Then, the normal curvature of *P* with respect to $q_{i}$ is estimated as follows:

(1) $$k_{n}^{i}=-\frac{\sin\beta }{|pq_{i}|\sin\alpha }$$

where *α* is the angle between vectors *N* and $pq_{i}$, *β* is the angle between vectors *N* and $M_{i}$, and $k_{n}^{i}$ denotes the normal curvature of the normal intercept line corresponding to the ith nearest neighbor point.

The following equation gives the approximation of Equation (1):

(2) $$k_{n}^{i}=-\frac{n_{xy}}{\sqrt{n_{xy}^{2}+n_{zi}^{2}}.\sqrt{x_{i}^{2}+y_{i}^{2}}}$$

where $n_{xy}=\frac{x_{i}\cdot n_{xi}+y_{i}\cdot n_{yi}}{\sqrt{x_{i}^{2}+y_{i}^{2}}}$.

As shown in Figure 3, let $e_{1}$ and $e_{2}$ be the principal directions corresponding to the principal curvature of point *P*. Supposed that θ is the angle between the *X*-axis in the local coordinate system and the principal direction corresponding to the maximum principal curvature $e_{1}$, $Q_{i}$ is the projection point of the nearest neighbor $q_{i}$ on the tangent plane *S*, and $\theta_{i}$ is the angle between vector $P\theta_{i}$ and the *X*-axis in the local coordinate system.

According to the Euler Equation, the normal curvature and the principal curvature are related as follows:

(3) $$k_{n}^{i}=k_{1}cos(\theta_{i}+\theta )+k_{2}sin^{2}(\theta_{i}+\theta ),(i=1,2,\;\ldots ,m)$$

The task can be written as an optimization problem:

(4) $$\underset{k_{1},k_{2},q}{\min}\sum_{i=0}^{m}{\left[k_{1}cos^{2}(\theta_{i}+\theta )+k_{2}sin^{2}(\theta_{i}+\theta )-k_{n}^{i}\right]}^{2}$$

Equation (4) can be translated into the following least squares problem.

(5) $$\underset{\mu }{\min}||\underset{m,3}{M}\underset{3,1}{\mu }-\underset{m,1}{r}|{|}^{2}$$

In Equation (5): $M_{m\times 3}=\left[\begin{array}{ccc} \cos^{2}\theta_{1} & 2\cos\theta_{1}\sin\theta_{1} & \sin^{2}\theta_{1}\\ \vdots & \vdots & \vdots \\ \cos^{2}\theta_{i} & 2\cos\theta_{i}\sin\theta_{i} & \sin^{2}\theta_{i}\\ \vdots & \vdots & \vdots \\ \cos^{2}\theta_{m} & 2\cos\theta_{m}\sin\theta_{m} & \sin^{2}\theta_{m} \end{array}\right]$; $r_{m\times 1}=\left[\begin{array}{c} k_{n}^{1}\\ \vdots \\ k_{n}^{i}\\ \vdots \\ k_{n}^{m} \end{array}\right]$; $\mu =(A,B,C{)}^{T}$; $A=k_{1}\cos^{2}\theta +k_{2}\sin^{2}\theta$; $B=(k_{2}{-k}_{1})\cos\theta \sin\theta$; $C=k_{1}\sin^{2}\theta +k_{2}\cos^{2}\theta$.

After the least squares fit of Equation (5), estimates of μ can be obtained accordingly, and using the derived values of *A*, *B*, and *C*, the Weingarten matrix can be inferred as follows:

$$W=\left[\begin{array}{cc} A & B\\ B & C \end{array}\right]=\left[\begin{array}{cc} k_{1}\cos^{2}\theta +k_{2}\sin^{2}\theta & (k_{2}-k_{1})\cos\theta \sin\theta \\ (k_{2}-k_{1})\cos\theta \sin\theta & k_{1}\sin^{2}\theta +k_{2}\cos^{2}\theta \end{array}\right]\;=\left[\begin{array}{cc} \cos\theta & \sin\theta \\ -\sin\theta & \cos\theta \end{array}\right]\left[\begin{array}{cc} k_{1} & 0\\ 0 & k_{2} \end{array}\right]\left[\begin{array}{cc} \cos\theta & -\sin\theta \\ \sin\theta & \cos\theta \end{array}\right]$$

It can be concluded that the principal curvature points $k_{1}$ and $k_{2}$ are the eigenvalues of the matrix *W.*

From the above, it can be seen that the points satisfying $k_{1}=k_{2}\neq 0$ are identified as spherical points, but due to the measurement error of the point cloud, the spherical spacing error, and the existence of local fitting error, the derived principal curvature value will deviate from the actual value. The values of $k_{1}$ and $k_{2}$ are rarely guaranteed to be exactly equal. Therefore, when the absolute value of their difference Δ does not exceed some limit ξ, the point can be recognized as a spherical point. However, differences in the radii of different target spheres will lead to differences in the curvatures of the respective spherical points. In the case that the radii of target spheres in the scene are unknown and different, it is not suitable to choose a constant value of ξ.

This paper is designed to identify spherical points by the relative deviation of the principal curvature $\partial \Delta =\frac{\Delta }{H}$, where *H* is the mean curvature, $H=\frac{k_{1}+k_{2}}{2}$, and $\Delta =|k_{1}-k_{2}|$. A point in the point cloud is identified as a spherical point when it satisfies the following two conditions.

Condition 1: ∂Δ<α, where α is the critical value, which is set to 0.4 in the experiments described in this paper.

Condition 2: $k_{1}=k_{2}>0$, and it is possible to exclude plane points and hyperbolic points.

#### 2.2.2. Total Least Squares Algorithm-Corrected Sphere Parameters

The set of interior points corresponding to the optimal model parameters M* is obtained: $(x_{i},y_{i},z_{i}),i=1,2,\cdots ,n$, and the spatial spherical equation is established as:

(6) $$(x-a_{0}{)}^{2}+(y-b_{0}{)}^{2}+(z-c_{0}{)}^{2}=r^{2}$$

where $a_{0}$, $b_{0}$, and $c_{0}$ are the coordinates of the center of the sphere, and *r* is the radius of the sphere. Considering that there are errors in the three directions *x*, *y*, and *z*, let $v_{x}$, $v_{y}$, and $v_{z}$ be the error correction numbers in the three directions *x*, *y*, *z*, respectively. We can rewrite the spherical equation as follows:

(7) $$(x-v_{x}{)}^{2}+{\left(y-v_{y}\right)}^{2}+{\left(z-v_{z}\right)}^{2}=2(x-v_{x})a_{0}+2(y-v_{y})b_{0}+2(z-v_{z})c_{0}+r^{2}-a_{0}^{2}-b_{0}^{2}-c_{0}^{2}$$

After rearranging Equation (7), the following equation is obtained.

(8) $$\underset{n,1}{y}+e_{y}=(\underset{n,m}{A}+\underset{n,m}{E_{A}})\underset{m,1}{X}$$

$E_{A}$ and $e_{y}$ in Equation (8) denote the errors of the coefficient matrix *A* and the observation matrix y, respectively.where $y_{n\times 1}=\left[\begin{array}{c} x_{1}^{2}+y_{1}^{2}+z_{1}^{2}\\ x_{2}^{2}+y_{2}^{2}+z_{2}^{2}\\ \vdots \\ x_{n}^{2}+y_{n}^{2}+z_{n}^{2} \end{array}\right]$; $A_{n\times 4}=\left[\begin{array}{cccc} 2x_{1} & 2y_{1} & 2z_{1} & 1\\ 2x_{2} & 2y_{2} & 2z_{2} & 1\\ \vdots & \vdots & \vdots & \vdots \\ 2x_{n} & 2y_{n} & 2z_{n} & 1 \end{array}\right]$; $e_{n\times 1}=\left[\begin{array}{c} v_{x1}^{2}+v_{y1}^{2}+v_{z1}^{2}\\ v_{x2}^{2}+v_{y2}^{2}+v_{z2}^{2}\\ \vdots \\ v_{xn}^{2}+v_{yn}^{2}+v_{zn}^{2} \end{array}\right]$;

$E_{A_{n\times 4}}=\left[\begin{array}{cccc} 2v_{X1} & 2v_{y1} & 2v_{z1} & 1\\ 2v_{X2} & 2v_{y2} & 2v_{z2} & 1\\ \vdots & \vdots & \vdots & \vdots \\ 2v_{Xn} & 2v_{yn} & 2v_{zn} & 1 \end{array}\right]$;$X_{4\times 1}=\left[\begin{array}{c} a_{0}\\ b_{0}\\ c_{0}\\ r^{2}-a_{0}^{2}-b_{0}^{2}-c_{0}^{2} \end{array}\right]$.

It is generally solved by the singular value decomposition of the matrix. Firstly, singular value decomposition of the augmented matrix $\left[\begin{array}{cc} A & y \end{array}\right]$ is performed:

(9) $$\left[\begin{array}{cc} A & y \end{array}\right]=U\Sigma V^{T}$$

The resulting model parameter estimation is obtained as:

(10) $$X=-\frac{1}{v_{n+1,n+1}}{\left[v_{1,n+1},\cdots ,v_{n,n+1}\right]}^{T}$$

According to Equation (10), the spherical target parameter $X={\left[\begin{array}{cccc} a_{0} & b_{0} & c_{0} & r^{2}-a_{0}^{2}-b_{0}^{2}-c_{0}^{2} \end{array}\right]}^{T}$ is the optimal target estimate.

The PC-RANSAC point cloud sphere detection algorithm exploits the properties of the differential geometry of spherical point clouds. It uses point cloud principal curvature constraints to improve the quality of RANSAC sample points. The current optimal set of interior points obtained at the termination of the iteration is also corrected by the total least squares algorithm. This method reduces the influence of point cloud data coefficient matrix and observation vector errors on the fitted spherical surface results. The following experiments illustrate the effectiveness and practicality of the method.

## 3. Experimental Results and Analysis

The experimental operating environment was an Intel Core 2.4 GHz CPU and MATLAB 2020 platform. Numerous experiments with synthetic and real data were performed to validate the proposed method. Efficiency and accuracy comparisons and large-scale measurement experiments were carried out. The detection efficiency and accuracy comparisons were divided into a simulation data experiment and an actual acquisition standard ball experiment.

### 3.1. Detection Efficiency and Accuracy Verification Experiments

#### 3.1.1. Simulation Experiments

**Figure 4:**
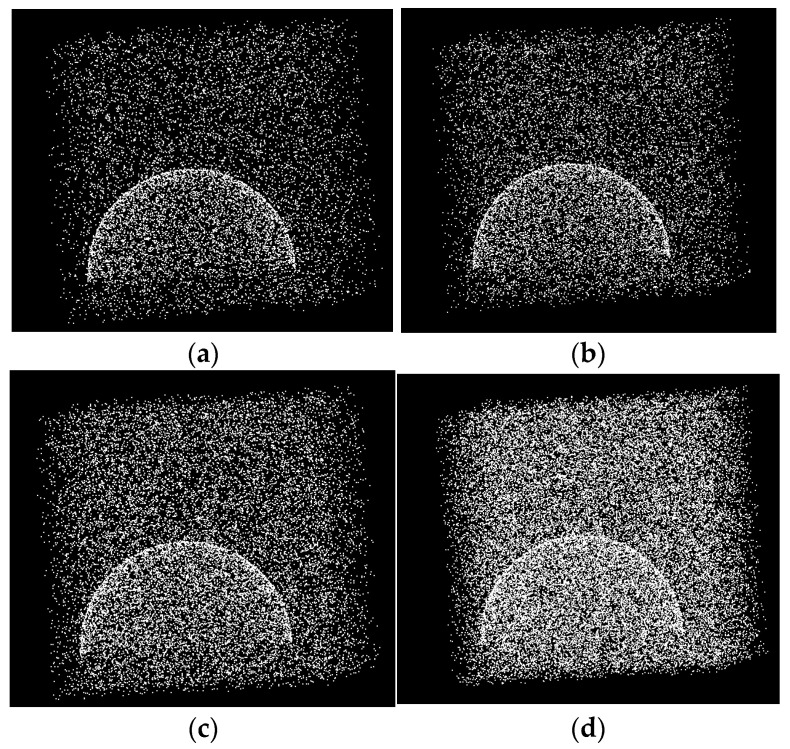
Point cloud of noisy spherical surface at different scales. (**a**) The proportion of the number of point clouds to noise points in the spherical model is 10%; (**b**) the proportion of the number of point clouds to noise points in the spherical model is 20%; (**c**) the proportion of the number of point clouds to noise points in the spherical model is 30%; (**d**) the proportion of the number of point clouds to noise points in the spherical model is 40%.

Because the spherical target captured by the surface structured light camera is usually below but close to the hemisphere, a standard hemispherical surface point cloud of 3000 points was generated by MATLAB. The coordinates of the center of the sphere were (20, 30, 40), and the radius of the sphere was 15. The noise in the point cloud was randomly generated, and the ratio of the number of spherical model point clouds to the number of noise points was set as W. Point clouds with W = 10% to W = 40% (with an interval of 10%) were generated respectively, which are called noisy spherical point clouds. The noise-laden spherical point cloud is shown in Figure 4.

**Figure 5:**
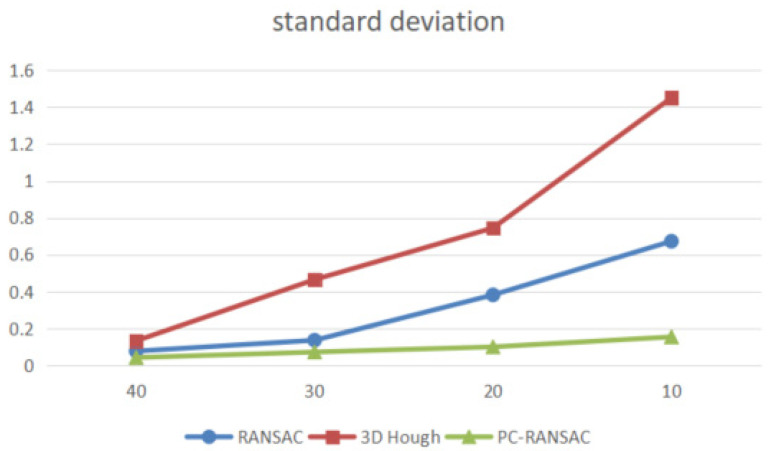
The standard deviation of sphere parameters estimated by different methods of simulation data.

Spherical point detection was performed on the simulated point cloud data by the RANSAC algorithm, 3D Hough algorithm, and PC-RANSAC algorithm. The experimental results are given as the mean and standard deviation of the sphere center coordinates and sphere radius after repeating 20 independent experiments. In this paper, the standard deviations of the detected center coordinates and radius of the spherical model from the actual values are used to represent the accuracy of the algorithm. The standard deviation is a measurement concept of the degree of dispersion of a set of values from the mean value. The standard deviation represents the magnitude of the calculated accuracy. The standard deviation calculation formula is shown in Equation (11), and the experimental results are shown in Table 1 and Figure 5.

(11) $$s=\sqrt{\frac{{\left(\bar{x}-x\right)}^{2}+{\left(\bar{y}-y\right)}^{2}+{\left(\bar{z}-z\right)}^{2}+{\left(\bar{r}-r\right)}^{2}}{4}}$$

where $\left(\bar{x},\bar{y},\bar{z}\right)$ and $\bar{r}$ are the estimated sphere center coordinates and radius of the spherical surface, respectively.

Table 1 shows that the detection time of the three algorithms starts to increase as the proportion of noise in the point cloud increases. The detection time of the 3D Hough algorithm increases more, the RANSAC algorithm is better than the 3D Hough algorithm in terms of speed, and the detection time of the PC-RANSAC algorithm is always less than that of the traditional RANSAC algorithm. Figure 5 shows that when the noise is low, the accuracy of the three methods in detecting the spherical model is not much different. However, as the noise increases, the accuracy of the RANSAC algorithm becomes better than that of 3D Hough. The accuracy of the RANSAC algorithm is better than that of the 3D Hough algorithm in detecting spherical targets in point clouds containing noise, and the results of the PC-RANSAC algorithm are closer to the real values and are always much better than those of the RANSAC algorithm and 3D Hough algorithm. The experimental results show that the PC-RANSAC algorithm can improve the efficiency and accuracy of the traditional RANSAC algorithm for detecting spherical surfaces with better robustness and accuracy.

#### 3.1.2. Standard Ball Experiment

**Figure 6:**
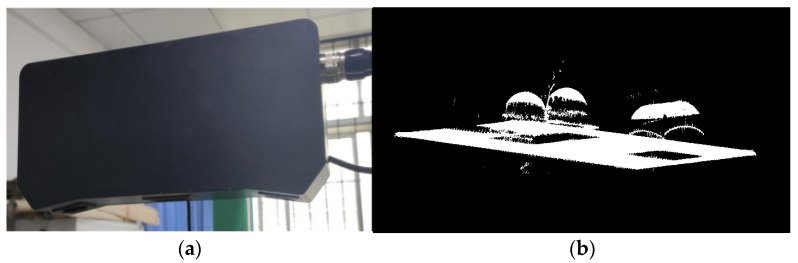
Experimental apparatus and scanning results. (**a**) Three-dimensional scanner; (**b**) actual scanned point cloud.

In this experiment, the point cloud data of standard ball and shaft parts were obtained by a XUNHENG 3D scanner, as shown in Figure 6a. The scanner performs photographic smooth surface scanning of the structure, and its adaptive range is from 40 mm to 2000 mm. Its scanning range is 200 mm, and its accuracy is 0.04 mm. In the original point cloud data, there are two ceramic standard balls and a shaft part. The diameter of the two standard balls is 30 mm, and the center distance of the two balls is 60 mm, as shown in Figure 6b. The original number of point clouds was 1 million, and after voxel downsampling, the number dropped to 82,340. The standard balls were measured using a global classic SR 05-07-05 CMM from HEXAGON. The diameters of the two standard balls were measured to be 30.004 mm and 30.005 mm, respectively, and the center distance of the two standard balls was 60.010 mm. Since the above experiment confirmed that the RANSAC algorithm outperforms the 3D Hough algorithm in detecting spherical surfaces in point clouds containing noise, a comparison experiment was conducted using RANSAC and PC-RANSAC to detect spherical surfaces.

**Figure 7:**
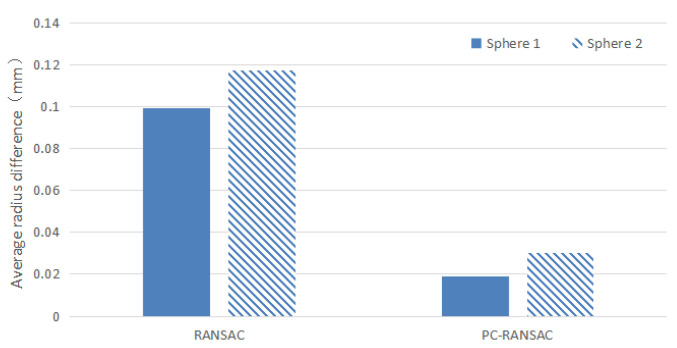
The average radius difference obtained by each algorithm in detecting the spherical surface.

To verify the robustness of this algorithm, a more relaxed threshold was chosen. The number of iterations in the experiment was set at k = 20,000, and the distance threshold was chosen to be 0.4. The experimental results were obtained after repeating 20 independent experiments to detect the spherical surface, as shown in Table 2; the sphere center distance between the two spherical surfaces, as shown in Table 3; and the average radius difference between the spherical surfaces, as shown in Figure 7. The total time required to detect the spherical surface is shown in Table 4.

As shown in Figure 7 and Table 3 and Table 4, the PC-RANSAC algorithm detected the average sphere center distance of the standard ball as 59.990 mm with a detection time of 4.62 s. The RANSAC algorithm detected the average sphere center distance of the standard ball as 59.587 mm with a detection time of 8.58 s. The experimental results show that the PC-RANSAC algorithm can effectively detect the spherical surface in the point cloud model, and the detection accuracy and speed are improved compared to the traditional RANSAC. The experimental results fully demonstrate the effectiveness and feasibility of the method.

### 3.2. Large-Scale Measurement Experiment

In order to verify the practicality of the proposed algorithm in large-scale coordinate conversion, the experiment used a spherical target and a robotic arm-driven 3D scanner as the measurement tool and a standard rod as the measurement object. A standard sphere fixed the standard rod fitting at both ends of the aluminum profile, and the sphere center distance measured by the articulated arm was used as the standard value. The sphere center distance of the two ends of the standard rod was obtained as 861.890 ± 0.008 mm, and the two sphere diameters were 30.007 ± 0.025 mm and 29.990 ± 0.025 mm, respectively.

**Figure 8:**
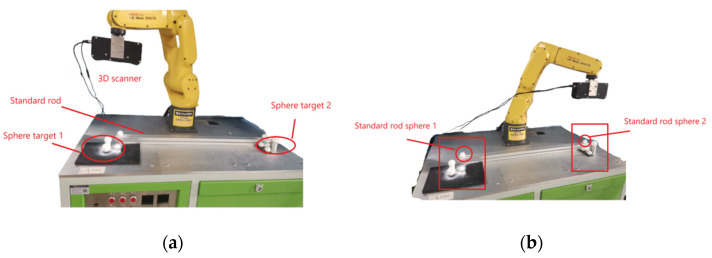
Coordinate unification experiment diagram: (**a**) measurement of target 1 and standard rod sphere 1; (**b**) measurement of target 2 and standard rod sphere 2.

The experimental procedure was as follows: (1) The positions of the two sets of targets were calibrated with the articulated arm before the measurement; the target positions are shown in the boxed parts in Figure 8a, and their measurement data are shown in Table 5.

(2) The standard rod fitting was placed on the horizontal surface of the adjacent target and fixed. Standard rod sphere 1 and target 1 and standard rod sphere 2 and target 2 were each scanned by operating the 3D scanner driven by the robotic arm, as shown in the square boxed area in Figure 8b. The improved RANSAC algorithm was used to automatically detect the target sphere point cloud and standard rod sphere point cloud in the point cloud data and fit them to obtain each sphere parameter. The measured data are shown in Table 6.

(3) For coordinate unification of point cloud data using the coordinates of the same spherical target in different measurement coordinate systems, the RT matrices of targets 1 and 2 converted to the joint arm coordinate system were obtained.

The RT matrix of target 1:$R_{1}=\left[\begin{array}{ccc} 0.929 & -0.331 & -0.166\\ 0.180 & 0.013 & 0.984\\ -0.323 & -0.944 & 0.072 \end{array}\right]$, $T_{1}=\left[\begin{array}{c} 65.718\\ 431.829\\ -548.694 \end{array}\right]$.

The RT matrix of target 2:$R_{2}=\left[\begin{array}{ccc} 0.922 & -0.349 & -0.167\\ 0.181 & 0.007 & 0.984\\ -0.342 & -0.937 & 0.069 \end{array}\right]$, $T_{2}=\left[\begin{array}{c} 328.124\\ 431.778\\ 259.960 \end{array}\right]$.

**Figure 9:**
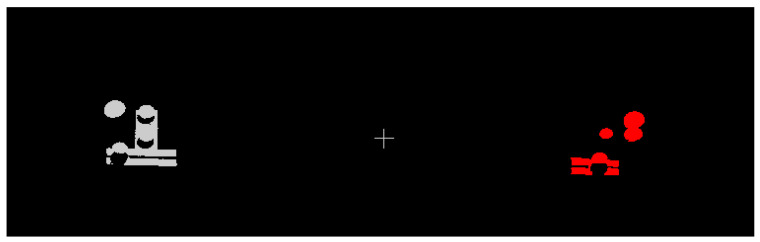
The effect of point cloud data coordinate unification.

(4) The rotation translation matrix of the point cloud data of targets 1 and 2 derived from step (3) converted and unified the two parts of the point cloud data into the joint arm coordinate system. The unified point cloud data are shown in Table 7, and the unified effect is shown in Figure 9.

(5) As shown in Table 7, the spherical surface was detected by the improved RANSAC algorithm, and the length of the sphere distance between the two ends of the standard rod obtained by coordinate unification is 861.865 mm. The experiment was repeated ten times with the mobile robot, and the measured value of the joint contact arm was used as the real value of the sphere distance. The single measurement result was compared with the real value, and the experimental results and data are shown in Table 8.

After the point cloud data coordinates were unified, the sphere distance data between the two ends of the standard rod were obtained, as shown in Table 8. The maximum deviation was 0.097 mm, the minimum deviation was 0.018 mm, and the average was 861.876 mm. The standard deviation was 0.0481 mm, and the standard uncertainty of the measurement mean was 0. 01521 mm. With degrees of freedom v = 9 and confidence probability p = 95%, querying the t-distribution table yielded k = 2.821, so the mean extended uncertainty U = 0.045 mm. Then, the measurement result of the ball center distance after coordinate unification was 861.876 ± 0.045 mm. The results show the practicality of the proposed algorithm in large-scale coordinate unification.

According to the above experiments, the PC-RANSAC algorithm is better than the traditional algorithm and 3D Hough algorithm in terms of detection accuracy and speed. The PC-RANSAC algorithm uses the point cloud principal curvature to constrain the selection of sample points. The efficiency of the traditional RANSAC algorithm is improved, so the algorithm running time can be shortened. The PC-RANSAC algorithm also uses the total least squares algorithm to optimize the interior point set of the current optimal sphere obtained by fitting. It can reduce the influence of the coefficient matrix and observation vector error of the point cloud data on the sphere fitting result so that it can improve the accuracy of fitting sphere parameters.

## 4. Conclusions

This paper proposes a novel method to automatically detect sphere targets in point clouds and improve the accuracy of estimating sphere parameters. The main contributions and novelty of this paper are as follows:

(1) We propose an improved RANSAC point cloud spherical target detection and parameter estimation method based on principal curvature constraint. The method applies to the automatic extraction of spherical targets when the coordinates of large-scale combined measurements are unified. The algorithm improves the iteration efficiency by constraining the sample point quality through the principal curvature. Considering the errors in both the coefficient matrix and observation matrix when fitting the point cloud data, the method uses the total least squares algorithm to optimally estimate the sphere parameters.

(2) Experimental results show that this method can automatically detect spherical objects in point clouds. The experimental results show that the proposed method has better detection accuracy and detection speed than the traditional RANSAC algorithm. The method was also applied to the coordinate unification of large-scale combined measurements. The practicability of the proposed algorithm is proved.

## Figures and Tables

**Table 1:** Sphere detection results of simulation data for each method.

W	Fitting Method	Sphere Parameter (mm)				Time (s)
		*x*	*y*	*z*	*r*	
40	RANSAC	19.951	30.017	39.907	14.890	5.24
	3D Hough	20.158	30.087	39.887	14.841	10.25
	PC-RANSAC	20.158	29.989	40.059	14.947	3.85
30	RANSAC	20.120	29.881	40.156	15.145	9.35
	3D Hough	20.461	29.438	40.379	15.438	15.24
	PC-RANSAC	20.114	29.956	39.979	14.925	4.22
20	RANSAC	19.819	29.776	40.294	14.575	13.45
	3D Hough	20.755	29.312	39.324	14.152	17.35
	PC-RANSAC	19.924	30.018	39.857	14.883	5.31
10	RANSAC	20.855	29.437	39.322	14.447	15.45
	3D Hough	21.755	28.532	41.204	13.682	20.54
	PC-RANSAC	20.149	29.836	39.843	14.855	7.53

**Table 2:** Sphere detection results of the data for each method.

Sphere	Detection Method	Estimated Sphere Parameters (mm)			
		*x*	*y*	*z*	*r*
Sphere 1	RANSAC	−47.625	15.835	587.507	14.903
	PC-RANSAC	−47.387	16.021	587.681	15.021
Sphere 2	RANSAC	−45.029	−43.562	583.516	14.885
	PC-RANSAC	−45.352	−43.806	583.752	14.972

**Table 3:** Average sphere center distance detected by each algorithm.

Detection Method	Distance between the Centers of the Two Spheres (mm)
RANSAC	59.587
PC-RANSAC	59.990

**Table 4:** Detection time of spherical surface for each algorithm.

Detection Method	Time (s)
RANSAC	8.58
PC-RANSAC	4.62

**Table 5:** Target ball center values for articulated arm calibration.

Group	Serial Number	The Coordinates of Sphere Centers (mm)		
		*x*	*y*	*z*
Target 1	Sphere 1	386.703	−15.863	239.877
	Sphere 2	425.466	−10.260	265.832
	Sphere 3	368.809	−10.346	285.568
Target 2	Sphere 1	116.247	−15.956	−561.551
	Sphere 2	126.015	9.198	−616.762
	Sphere 3	76.307	−8.218	−600.384

**Table 6:** Sphere center values of the scanner’s detection target.

Group	Serial Number	The Coordinates of Sphere Centers (mm)		
		*x*	*y*	*z*
Target 1	Sphere 1	386.703	−15.863	239.877
	Sphere 2	425.466	−10.260	265.832
	Sphere 3	368.809	−10.346	285.568
Target 2	Sphere 1	116.247	−15.956	−561.551
	Sphere 2	126.015	9.198	−616.762
	Sphere 3	76.307	−8.218	−600.384
Standard rod sphere	Sphere 1	78.436	−33.811	−437.573
	Sphere 2	70.601	−22.050	−435.581

**Table 7:** Measurement data after coordinate unification.

Group	Serial Number	The Coordinates of Sphere Centers (mm)		
		*x*	*y*	*z*
Target 1	Sphere 1	386.729	−15.868	239.832
	Sphere 2	425.442	−10.260	265.833
	Sphere 3	368.806	−10.34	285.612
Target 2	Sphere 1	116.37	−15.935	−561.539
	Sphere 2	126.056	9.176	−616.689
	Sphere 3	76.143	−8.217	−600.469
Standard rod sphere	Sphere 1	485.284	15.305	234.505
	Sphere 2	210.394	15.931	−582.346

**Table 8:** Data of ball center distance after coordinate unification.

Serial Number	Distance between the Centers of the Two Spheres (mm)
1	861.865
2	861.793
3	861.926
4	861.966
5	861.864
6	861.926
7	861.842
8	861.867
9	861.836
10	861.872

## Data Availability

Not applicable.
